# Diagnosis and Management of Pediatric Non-Alcoholic Fatty Liver Disease: An Overview

**DOI:** 10.3390/metabo15120792

**Published:** 2025-12-12

**Authors:** Dalia Dop, Vlad Pădureanu, Rodica Pădureanu, Carmen Elena Niculescu, Ștefan Adrian Niculescu, Iulia Rahela Marcu

**Affiliations:** 1 Department of Pediatrics, University of Medicine and Pharmacy of Craiova, 200349 Craiova, Romania; dalia.dop@umfcv.ro (D.D.); carmen.niculescu@umfcv.ro (C.E.N.); 2 Department of Internal Medicine, University of Medicine and Pharmacy of Craiova, 200349 Craiova, Romania; 3 Department of Orthopedics, University of Medicine and Pharmacy of Craiova, 200349 Craiova, Romania; stefan.niculescu@umfcv.ro; 4 Department of Physical and Rehabilitation Medicine, University of Medicine and Pharmacy of Craiova, 200349 Craiova, Romania; rahela.marcu@umfcv.ro

**Keywords:** NAFLD, children, metabolic dysfunction, liver fibrosis, gut microbiota

## Abstract

Non-alcoholic fatty liver disease (NAFLD) is the most common pediatric chronic liver disease worldwide, with an increasing prevalence, mainly due to the increase in childhood obesity and sedentary lifestyle. The pathogenesis of NAFLD is multifactorial, but the mechanisms by which the factors involved, namely the genetic, intrauterine and environmental factors responsible for its onset and progression to NASH, are not fully known. Children with NAFLD are usually asymptomatic or show nonspecific symptoms, and NAFLD is generally diagnosed incidentally by screening tests in overweight or obese children. NAFLD is associated with severe metabolic deficiencies that may progress to cirrhosis and hepatocellular carcinoma, with the consequent need for liver transplantation. Current treatment of NAFLD in children consists of lifestyle changes to decrease caloric intake and increase physical activity, with no currently approved pharmacological medication for the pediatric population. Although pediatric studies that focus on alternative treatments targeting key pathogenic factors are promising, no pharmacological agent is currently approved for children, validated non-invasive fibrosis biomarkers remain limited, and long-term outcome data are scarce. Further validation through large prospective pediatric cohorts and phase III trials is urgently needed.

## 1. Introduction

Non-alcoholic fatty liver disease (NAFLD) is defined by fatty liver infiltration of more than 5% of hepatocytes, in the absence of excessive alcohol consumption or evidence of viral, metabolic, autoimmune or drug-induced liver disease, and it is assessed by liver biopsy [1]. NAFLD is the most common cause of chronic hepatitis in children and it encompasses a spectrum of diseases, from simple hepatic steatosis to nonalcoholic steatohepatitis (NASH) which may at some point progress to fibrosis, cirrhosis and liver failure and implicitly the need for life-sustaining liver transplantation [2,3].

The prevalence of NAFLD in the pediatric population is increasing and is estimated at 13% (9.8% adjusted), [4] with an age-dependent increase that correlates with increasing life span, increasing obesity and diabetes mellitus among children.

The pathogenesis of pediatric NAFLD involves a multifactorial interaction between the genetic factor and epigenetic influences in the prenatal and postnatal period, with a strong psychosocial impact, and it is different from adult pathology [5,6].

NAFLD in children is associated with several extrahepatic manifestations, including insulin resistance, hyperlipidemia, polycystic ovary syndrome or obstructive sleep apnea [7,8].

Although liver histology is considered the gold standard for the assessment of NAFLD, performing a liver biopsy is not feasible in children, so various scoring systems have been developed in order to quantify and monitor fatty changes and liver fibrosis, including laboratory data, radiologic explorations and novel biomarkers [4].

The current standard treatment of NAFLD in children is lifestyle change through diet and physical activity for weight loss [9].

Although dysfunction-associated steatotic liver disease (MASLD) has recently been proposed as a new nomenclature, indicating its association with metabolic dysfunction, we have compiled the scientific literature on the pathogenesis, diagnosis, and treatment of pediatric NAFLD, which was the predominant terminology in the literature prior to this revision.

Diagnosing NAFLD/MASLD in clinical practice remains complex, but it is a diagnosis that should not be overlooked because it can increase the risk of cardiovascular disease, metabolic disorders, and mortality in adulthood [4].

This article aims to summarize the current status of pediatric NAFLD, the diagnostic methods and future directions of intervention and therapeutic goals.

## 2. Materials and Methods

An extensive search of the literature was conducted using the Scopus, PubMed, and Web of Science databases to identify studies addressing recent advances in the pathogenesis, prevention, diagnosis, and treatment of pediatric patients with NAFLD. The search was conducted between January 2025 and June 2025, using keywords such as “non-alcoholic steatohepatitis”, “non-alcoholic fatty liver disease in children”, “fatty liver in children”, and “fatty liver disease associated with metabolic dysfunction.”

The search strategy focused on guidelines, original clinical studies, systematic reviews, and was limited to English-language publications and articles referring to children under the age of 18. To refine the search, which initially generated 623 records, we used Boolean operators and truncation symbols. After applying filters for publication date and English language, the number of records was reduced to 320.

During the first screening, keywords, titles, and abstracts were searched, and publications that did not meet the above search criteria were eliminated. This selection reduced the number of articles to 185, which were considered suitable for a full analysis. Subsequently, a detailed evaluation of these full texts was performed to confirm their relevance to our objective, and they were classified into categories according to pathogenesis, clinical sign, treatment, or article type (clinical study or systematic review), excluding studies with unclear methodologies or those limited to case reports or conference abstracts, resulting in 104 records.

**Figure 1:**
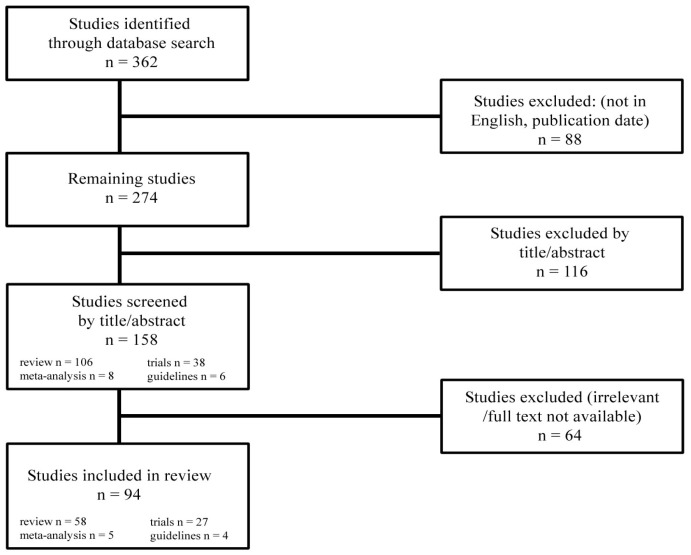
Study selection.

We included studies that referred to children diagnosed with NAFLD, provided clear diagnostic criteria and outcome measures, and were published as original research, systematic reviews, or meta-analyses (Figure 1).

## 3. Epidemiology

NAFLD has been reported in people of all ethnicities and races, but it is more common in the Hispanic or Asian American population and less common in African Americans, although the latter often present more risk factors for NAFLD—obesity, insulin resistance and type 2 diabetes [3,10]. Ethnic differences are due to dissimilar socio-economic and environmental factors, implicitly the type of diet or physical exercises or a higher rate of insulin resistance reported for an equivalent body mass index (BMI) [11]. As far as the age is concerned, although it can also be present in younger children, with documented cases under 2 years of age [10], NAFLD has an increased incidence in adolescents both due to increased obesity among them but also due to increased insulin resistance and increased levels of sex hormones [3,12]. In terms of sex of the patients, there is a male predominance in obese adolescent patients with NAFLD, possibly due to the protective effect of estrogen hormones on the liver or the aggravating effect of androgen hormones [13,14,15].

The prevalence of pediatric NAFLD varies widely depending on the characteristics of the population, in particular lifestyle habits of the patients and the diagnostic method used to detect it, and is estimated to be between 3% and 10% Table 1 [3,16,17]. NAFLD is estimated to affect 34% of obese children aged 2–19 years and 10% of the general pediatric population [18]. NAFLD can occur in 8 to 16% of non-obese children, and the causes are visceral obesity in non-obese children as well as genetic and environmental factors [19,20].

A 2015 meta-analysis that included 74 publications identified a prevalence of pediatric NAFLD in Europe of 5.7% in the general population and 33% in the obese population, while in Asia the prevalence was 5.9% in the general population and 62.3% in the obese population. In North America, the prevalence was 6.5% in the general population and 39.2% in the obese population, in South America the prevalence was 25.1% in the general population and 17.1% in the obese population, and in the Middle East and North Africa it was 6.8% in the general population and 36.5% in the obese population [17].

## 4. Pathogenesis

**Figure 2:**
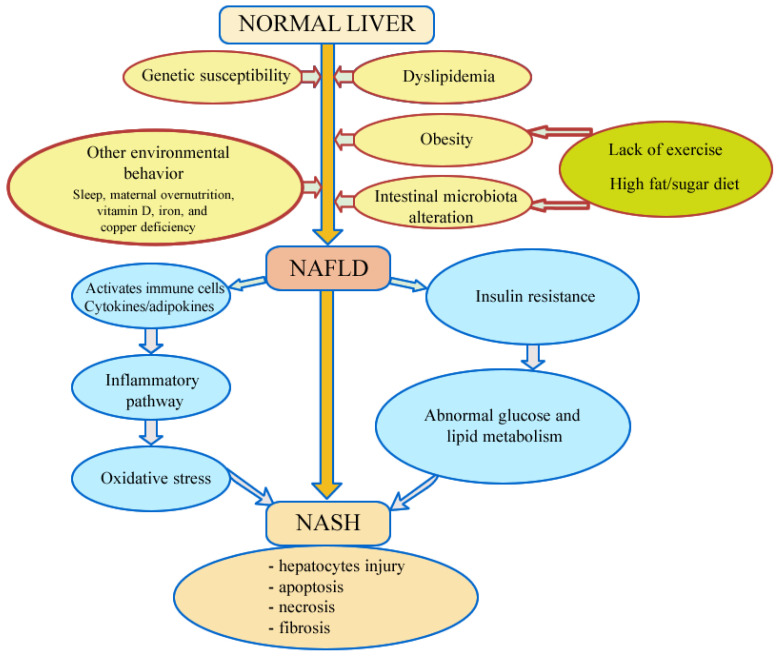
Pathomechanisms involved in development and progression of non-alcoholic fatty liver disease.

The pathogenesis of NAFLD is not yet fully elucidated, currently the “multiple hit” hypothesis is the accepted one; it considers multiple insults acting together: genetic and epigenetic factors, insulin resistance, hormones secreted from the adipose tissue, nutritional factors, gut microbiota [21]. Poor eating habits, along with other environmental factors, can lead to obesity in genetically predisposed individuals, with the proliferation of adipocytes, increased insulin resistance, and implicit changes in the intestinal microbiome. Excessive energy intake induces hypertrophy and hyperplasia of adipose tissue with the onset of obesity and subsequent development of systemic insulin resistance, which is the first step, in the development of NAFLD [3]. At the hepatic level, lipid accumulation resulting from the excessive influx of fatty acids from endogenous fat stores, decreased synthesis of apolipoprotein B-100, de novo hepatic lipogenesis and excessive dietary fat intake lead to the development of steatosis that characterizes NAFLD [5]. Subsequently, oxidative stress accounts for the progression to liver fibrosis. Reactive oxygen species (ROS) can induce hepatocellular injury by inactivating the glyceraldehyde-3-phosphate dehydrogenase, inhibiting the mitochondrial respiratory chain enzymes, inactivating the membrane sodium channels, followed by lipid peroxidation, production of proinflammatory cytokines and endotoxin-mediated activation of the innate immune response, contributing to hepatocellular injury and fibrosis [22]. In this pathogenic chain, altered intestinal flora leads to additional production of fatty acids in the intestine, increased permeability of the small intestine, and thus increased circulating levels of molecules that contribute to the activation of inflammatory pathways and the release of proinflammatory cytokines. NASH is characterized by inflammation, mitochondrial dysfunction, oxidative stress, and fibrosis [23] (Figure 2).

### 4.1. Insulin Resistance

Thus, the first stage consists of the intrahepatic accumulation of fatty acids, which is linked to insulin resistance and increases the susceptibility of hepatocytes to secondary injury (mitochondrial dysfunction, oxidative stress, overproduction and release of proinflammatory cytokines) [3].

Overeating causes the accumulation of liver fats that contribute to the production of most adipokines, such as leptin, resistin, adiponectin, tumor necrosis factor-alpha (TNF-alpha), and IL-6, which are involved in inducing insulin resistance and inflammation [21].

Leptin is an adipose-specific satiation hormone produced predominantly in adipocytes, that can regulate metabolism and satiation via the hypothalamus [24,25].

Adiponectin is an adipokine that has anti-inflammatory and insulin-sensitizing properties [26], while resistin is another adipokine that antagonizes the action of insulin, causing glucose intolerance; increased resistin is associated with insulin resistance [27]. Lifestyle changes may impact on adiponectin production, which is promising. TNF-alpha and IL-6 indirectly mediate lipolysis and increase hepatic fatty acid synthesis [28,29], and they are positively related to adiposity and are correlated with cardiovascular disease (CVD) risk factors and insulin resistance [30].

Fatty hepatocytes release large amounts of extracellular vesicles that transport bioactive molecules such as DNA, mRNA, proteins, and lipids to target cells, leading to the recruitment or activation of macrophages and silent hepatic stellate cells (HSCs), respectively, which cause inflammation and fibrogenesis [31,32]. Triglyceride (TG)-rich chylomicrons are mainly transported to peripheral tissues (80%), where via lipoprotein lipase (LPL), free fatty acids (FFA) are released and available for absorption [33].

In people with insulin resistance, insulin fails to effectively suppress the apolipoprotein C-III (ApoC-III), which is one of the most potent inhibitors of LPL, which inhibits LPL action in peripheral tissues and favors hepatic uptake of TG-rich chylomicron debris [24]. The depletion of adipocyte expandability can produce oxidative stress, lipotoxicity, and peripheral insulin resistance [33].

### 4.2. Oxidative Stress

The increased influx of free fatty acids into the liver overloads the mitochondria and leads to increased beta-oxidation, which reduces the availability of oxidized cofactors (NAD and FAD) and decreases the outflow from the respiratory chain, leading to electron accumulation, ROS production and cell damage. Oxidative changes in the respiratory complexes impair catalytic functions and cause mutagenesis of mitochondrial DNA, which further exacerbates the oxidative damage, leading to hepatocellular death and NASH progression [34].

Since insulin is the main inhibitor of cytochrome P450 4A (CYP4A), the enzyme involved in peroxisomal oxidation, the increased insulin resistance leads to increased oxidation and thus amplifies lipid peroxidation and the production of cytotoxic reactive oxygen species (ROS) [35]. Reactive oxygen species (ROS) cause the inhibition of mitochondrial respiratory chain enzymes; the inactivation of the membrane sodium channels and of the glyceraldehyde-3-phosphate dehydrogenase [33,36]. They diffuse into the extracellular space, can influence Kupffer cells and induce the nuclear factor pathway κB (NFκB), which causes the production of proinflammatory cytokines and fibrinogen and induces Fas ligand, contributing to hepatocellular injury and fibrosis [34].

Mitochondrial oxidative damage increases the tendency of mitochondria to release proteins from the intermembrane space into the cytosol through mitochondrial outer membrane permeabilization (MOMP), thereby activating the apoptotic mechanism of the cell [24,37].

This progression is influenced and maintained by complex interactions of the genetic, hormonal and environmental factors, in the pre- and postnatal period, as well as by links between different organs and tissues: the gut and the liver, adipose tissue and the pancreas [38].

Metabolic programming is influenced by prenatal factors predisposing to NAFLD: low birth weight, maternal body mass index, metabolic syndrome or gestational diabetes [39].

### 4.3. Genetic Susceptibility

Genes involved in lipid metabolism or inflammation may influence the onset of progressive liver disease, type 2 diabetes mellitus or hepatocellular carcinoma. Thus, the most well-known adiponutrin/patatin-like phospholipase domain-containing 3 (PNPLA3) gene, a variant in the patatin-like phospholipase domain-containing protein 3 (PNPLA3) gene, is associated with increased levels of the liver enzymes ALT and AST in young patients, fibrosis development and carcinoma risk, having a higher presence in the Hispanic community. The S453I allele of the PNPLA3 gene, which is protective, is found in African Americans and explains the lower prevalence of NAFLD in this community [40].

Lysosomal acid lipase (LAL) deficiency, which causes increased lysosomal cholesterol ester storage, has been observed in children with NAFLD, suggesting its involvement in disease progression [41,42]. A variant in the glucokinase regulatory protein (GCKR) gene has been associated with an increased rate of DNL in obese adolescents, while other genes such as apolipoprotein C3 (APOC3) or the genes involved in inflammation, oxidative stress and fibrogenesis, such as SOD2, are associated with NAFLD and severity of liver damage [18].

A minor allele in the human transmembrane 6 superfamily 2 (TM6SF2) gene has been associated with higher fibrosis and NAFLD activity score in children [43], and the involvement of the CB2 Q63R variant of the endocannabinoid system receptor was associated with the severity of inflammation (*p* = 0.002) and increased predisposition of these patients to develop liver injury [44].

### 4.4. The Role of Nutrients in Pediatric NAFLD

High-calorie diets enriched with fats and fructose, and sucrose can lead to NAFLD by hepatic accumulation of fatty acids, leading to deposition of visceral fat and abdominal obesity or favoring the development of systemic insulin resistance (IR) [24].

High blood glucose levels lead to the activation of carbohydrate response element-binding protein (ChREBP), which physiologically regulates de novo insulin-independent glycolysis and lipogenesis and causes liver fat accumulation.

Certain dietary sugars, particularly fructose, are suspected to contribute to the development and progression of NAFLD. Increased amounts of fructose in the diet come from fruits and vegetables with high fructan content (wheat, leek, garlic), honey, sugar additives (most commonly sucrose, fructose and corn syrup), beverages and processed foods. Substantial links have been shown between high fructose intake and obesity, dyslipidemia and insulin resistance [45].

The metabolism of fructose is relatively unregulated by insulin, as the metabolism is carried out by GLUT5 and not by the insulin-dependent transporters GLUT1 and GLUT4. In hepatocytes, fructose is converted mainly by fructokinase to fructose 6-phosphate, which is then hydrolyzed to fructose 1-6 bisphosphate by fructose aldolase, in order to enter the gluconeogenesis pathway [24]. There is an increase in AMP and uric acid, with hepatic depletion of ATP. Fructose does not cause an acute rise in insulin, but it causes the induction of several hepatic lipogenic enzymes (pyruvate kinase, NADP+-dependent malate dehydrogenase, acetyl CoA carboxylase, fatty acid synthetase, pyruvate dehydrogenase) and an increase in hepatic fat storage and VLDL production [46].

A longitudinal study from 2021 demonstrated an association between high intake of sugar-containing beverages during infancy and NAFLD in children, independent of the intake of sugar-containing beverage and body mass index (BMI) at school age [47].

Children with NAFLD and obesity exhibited an exaggerated metabolic response to fructose administration compared to normal-weight children; this effect was due to the fermentation of fructose to hydrogen by the intestinal bacterial flora or an upregulation of the fructose transporter GLUT5 in the intestinal epithelium [48]. Thus, ingested fructose may result in altered gut microbiota and increased intestinal permeability, leading to an increase in endotoxin permeation into the portal system due to increased permeability of tight junctions [49].

### 4.5. Intestinal Microbial Dysbiosis in Pediatric NAFLD

In NAFLD we have a lower microbial diversity and a weakened intestinal barrier, which exposes the host to bacterial components and causes the stimulation of immune defense pathways and the onset of inflammation [50].

Intestinal microorganisms produce pathogen-associated molecules—lipopolysaccharides/endotoxins—which are recognized by specific pattern recognition receptors (PRRs), including TLRs and NOD-like receptors, causing increased intestinal permeability that activates molecular mechanisms of the innate immune response and may act as a possible inducer of NAFLD progression [9,51].

In obese subjects, both qualitative and quantitative differences in the composition of the bacterial flora have been found, due to host-related factors (e.g., immune system response, associated diseases, diabetes) [52].

The gut microbial flora breaks down non-absorbable polysaccharides into short-chain fatty acids (SCFAs) such as propionate and butyrate, which induce leptin production and increase fatty acid oxidation, and into monosaccharides that activate the hepatic carbohydrate response element-binding protein (ChREBP), leading to fat accumulation and increased hepatic lipogenesis [49].

Short-chain fatty acids (SCFAs) also have the potential to reduce intestinal permeability and weaken immunity, but their roles in pediatric NAFLD are not yet well understood [18].

Fermentation of proteins by anaerobic gut bacteria produces branched-chain fatty acids (BCFAs) and volatile organic compounds (VOCs), such as indole or phenylacetate, which are toxic to the host and can aggravate chronic liver disease [53].

Fermentation of proteins and carbohydrates produces gaseous molecules, such as NO and hydrogen sulfide (H2S), which act as gasotransmitters and affect inflammation and vasoregulation [24].

Studies show that an important role in the pathogenesis of NAFLD is played by the farnesoid X receptor (FXR), a nuclear receptor preferentially activated by primary bile acids and expressed in the intestinal epithelium and liver, which has a role in protecting the integrity of the intestinal barrier, regulating carbohydrate, lipid and amino acid metabolism, and producing antimicrobial peptides [24]. Another important bile acid sensor is the G protein-coupled receptor TGR5, which is preferentially activated by secondary bile acids and plays a role in reducing proinflammatory responses in macrophages, inhibits NLRP3 inflammasome activation, and regulates eNOS activity in sinusoidal endothelial cells (SEC) [54].

An important role in host–microbiome interactions and NAFLD progression is also played by ethanol. Intestinal microbial breakdown of unabsorbed dietary sugars produces endogenous ethanol and may explain the increased blood alcohol levels detected in non-drinkers diagnosed with steatohepatitis [24]. In the liver, ethanol produces acetaldehyde, which in turn is oxidized to nontoxic acetate. Exposure of the intestinal mucosa to acetaldehyde may increase intestinal permeability by disrupting intercellular complexes [55].

There are studies showing that increased levels of blood ethanol in patients with NAFLD may result from impaired insulin-dependent alcohol dehydrogenase (ADH) activity in liver tissue, rather than from increased endogenous ethanol synthesis [56,57].

### 4.6. Obstructive Sleep Apnea

In a meta-analysis of children, short sleep duration was associated with an increased risk of obesity [58].

Disruption of the circadian rhythm is associated with significant morbidity and mortality, including metabolic syndrome by affecting several metabolic regulatory genes that are synchronized with the circadian clock [59].

Thus, in Kettner’s 2016 study, wild-type mice bred under conditions of chronic sleep deprivation developed hepatic steatohepatitis and fibrosis [60].

Obstructive sleep apnea (OSA) associated with obesity is also considered a risk factor for more severe NAFLD [61]. Several studies have shown that chronic hypoxia in OSA favors the occurrence of liver inflammation and fibrosis through several mechanisms, including the promotion of inflammatory cytokines in hepatocytes and macrophages by hypoxia-inducible factor and NFκB, which then modulate fibrogenesis and angiogenesis in Kupffer cells. Liver injury also occurs through intensification of oxidative stress by the process of ischemia–reperfusion injury [62].

According to a study conducted on adolescents, the association of obstructive sleep apnea and NAFLD led to an increase in the tendency toward fibrosis, due to the histological grade of inflammation and increased NAFLD activity score [63].

Studies have shown that the association between OSA and NASH severity correlates with impaired intestinal barrier function, leading to increased TLR-4-mediated liver susceptibility to endotoxemia [64].

### 4.7. Other Mechanism Involved

Trace elements such as copper and iron are essential in the protection against inflammation and peroxidation present in obesity through immunologic, regulatory and antioxidant functions. Thus, it has been shown that disturbances in the detoxification processes of copper and iron in the liver are associated with the development of NAFLD through oxidative stress [64].

An important role in the development of steatosis and its progression to non-alcoholic steatohepatitis is played by the ghrelin-ghrelin O-acyltransferase (GOAT) system which is involved in energy and lipid metabolism, insulin resistance, inflammation and apoptotic cell death [65]. The role of the ghrelin-GOAT system in the pathogenesis of NAFLD is a topic of interest to researchers, and the role of ghrelin in appetite regulation and energy metabolism is now recognized as a promising target for the treatment of obesity and NAFLD [66].

A growing body of evidence suggests that low vitamin D levels are strongly associated with obesity and NAFLD. Vitamin D has receptors expressed in a wide range of tissues including the liver and the immune system (macrophages, T and B cells, monocytes), and its anti-inflammatory, metabolic and anti-fibrotic properties provide plausible mechanisms by which it may influence the progression and severity of NAFLD [67].

A 2015 study in children showed that there is a high prevalence of vitamin D deficiency in children with biopsy-confirmed NAFLD, but no association was found between vitamin D deficiency and disease severity. Studies of NAFLD in adults show correlation between vitamin D deficiency and histologic severity, thus suggesting differences regarding the risk factors or consequences of NAFLD in children [68].

Among the major factors in the development of NAFLD in overweight or obese children is decreased physical activity. Thus, a study conducted in overweight/obese children showed that liver fat content measured by magnetic resonance imaging was lower in those with better cardiorespiratory and musculoskeletal fitness [69].

Of great importance in the pathogenesis of NAFLD is the psychosocial aspect, because children with NAFLD as a consequence of obesity have increased levels of psychological impairment, and on the other hand, psychological disorders may be associated with increased risk of obesity and NAFLD [70].

### 4.8. Maternal Overnutrition and NAFLD Developmental Programming

Studies show that maternal nutrition and obesity play an important role in the development of the metabolic syndrome, of insulin resistance and NAFLD in the offsprings. Longitudinal and cross-sectional studies have demonstrated that high or low birth weight is associated with higher odds of severe steatosis and fibrosis in adolescents with biopsy-confirmed NAFLD, independent of childhood BMI [71].

A 2009 study shows that the developing fetus is highly vulnerable to excess lipids, independent of maternal diabetes and/or obesity, and that exposure to it may increase the risk of pediatric NAFLD. Changing the maternal chronic high-fat diet (HFD) during a subsequent pregnancy to a low-fat diet improved fetal hepatic triglyceride levels and gluconeogenic gene expression, suggesting that maternal diet is a major risk factor for early-onset NAFLD [72].

Risk factors involved in the onset of NAFLD/MASLD in obese children include male gender, positive family medical history, adolescence, Hispanic ethnicity, intestinal dysbiosis, endocrine disorders caused by environmental factors, or the presence of genetic variants of the patatin-like phospholipase domain-containing protein 3 (PNPLA3), membrane-bound acyltransferase 7 (MBOAT7), glucokinase regulatory protein (GCKR), and transmembrane superfamily member 2 (TM6SF2) [11].

## 5. Clinical Signs

Children with NAFLD are generally asymptomatic at the time of diagnosis, older than 10 years of age, and overweight/obese. The presence of acanthosis nigricans, increased waist circumference and hepatomegaly are markers of insulin resistance, central/visceral obesity and liver damage, the combination of these signs indicating an increased risk of NAFLD. Patients present, in particular due to the association of obesity, a reduced tolerance to physical exertion, drowsiness, flatulence, muscle pain, psycho-emotional disturbances, social adjustment difficulties, thus contributing to a decreased quality of life in these patients [73].

In some cases, right upper quadrant pain may occur which may be attributable to liver capsule distension secondary to hepatic steatosis, leading to hepatomegaly and elevated liver enzymes [4].

Blood pressure assessment, control, and monitoring should be mandatory components of clinical management in children with NAFLD, because patients with non-alcoholic steatohepatitis (NASH) and/or obesity are at higher cardiovascular risk due to peripheral insulin resistance, oxidative stress, and systemic inflammation [24].

Signs of liver failure present in adults, such as jaundice, ascites, palmar erythema, encephalopathy, and abdominal wall angiomas are rarely seen in children. If the medical history and clinical examination show signs of hyperandrogenism (irregular menstrual cycles, acne, hirsutism), screening for polycystic ovary syndrome (PCOS) is recommended in adolescent girls with NAFLD [24].

## 6. Diagnosis

NAFLD is a diagnosis of exclusion that requires the presence of hepatic steatosis and the exclusion of other causes of hepatic steatosis besides NAFLD.

Although liver biopsy is the gold standard for confirming the diagnosis and staging NAFLD, its use in pediatric practice is limited due to its invasiveness and associated risks.

Liver biopsy, according to the recommendations of the North American Society for Pediatric Gastroenterology, Hepatology, and Nutrition (NASPGHAN), should be considered in patients at high risk for NASH and advanced fibrosis (elevated transaminases and splenomegaly) or in thin children with features that may indicate an alternative diagnosis [74]. In this context, increasing importance is being given to non-invasive strategies which can allow the assessment of the degree of liver damage. These include assessments based on serologic markers, modern imaging techniques such as transient elastography or MRI with quantification of lipid content, as well as emerging biomarkers with diagnostic and prognostic potential [24].

An essential component of laboratory assessment is designed to exclude other conditions with similar clinical manifestations. The selection of investigations is guided by the patient’s age, medical history, physical examination, and the presence of risk factors for NAFLD, including genetic, infectious, endocrine, or autoimmune etiologies [75].

## 7. Differential Diagnosis

The incidence of NAFLD has increased in recent years, mainly due to the rise in the prevalence of obesity in children. The diagnosis of NAFLD is one of exclusion, so it is essential to use an algorithm that takes into account the child’s age and is based on clinical characteristics and then blood tests; at a later stage, a liver biopsy should be considered.

Abnormal serum aminotransferases in overweight or obese children are not always diagnostic of NAFLD/NASH, and other causes must be ruled out, including muscle diseases or liver diseases. It is essential to identify conditions that can be treated with specific therapies, such as celiac disease, Wilson’s disease, inflammatory bowel disease, or autoimmune hepatitis [22].

## 8. Biochemical Assessment

Alanine aminotransferase (ALT) is the most commonly used biochemical marker in the evaluation of liver disease in children, but its lack of specificity requires the exclusion of other liver etiologies with similar manifestations. ALT reference values vary with age and sex [76], but an ALT value increased to more than twice the upper level after exclusion of other causes in overweight children older than 10 years of age is a strong indicator for NAFLD (88% sensitivity and 26% specificity) [74].

AST/ALT ratio values are usually subunitary but may progressively increase as liver fibrosis progresses. Aminotransferase levels may remain within normal ranges in a considerable percentage of children with NAFLD or even NASH, which does not exclude the presence of significant liver damage. Alkaline phosphatase and gamma-glutamyl transferase (GGT) may be slightly elevated [3], while serum bile acid (BA) concentrations are low in the early stages of NAFLD and increase progressively with worsening liver fibrosis. Given the association of elevated bile acid levels with cirrhosis in adults, monitoring them may represent a valuable non-invasive biomarker for the assessment of the progression of NAFLD in pediatric patients.

It is recommended to determine the glycemic parameters, to calculate the Homeostatic Model Assessment for Insulin Resistance (HOMA-IR), and to analyze the lipid profile for all children with suspected or confirmed diagnosis of NAFLD [24].

Serum values of albumin, bilirubin and platelets are within physiological limits unless the patient develops cirrhosis [3].

In some cases, autoantibodies such as antinuclear antibodies and anti-smooth muscle antibodies may be detected, which may be associated with more advanced forms of fibrosis [3].

Advanced biomarkers provide essential information about the progression and severity of liver damage. Cytokeratin 18 (CK-18) fragments, indicative of hepatocyte apoptosis, correlate the relationship between inflammation and disease progression, while markers of extracellular matrix remodeling (hyaluronic acid, tissue inhibitor of metalloproteinase-1 and amino-terminal propeptide of type III procollagen) reflect fibrosis stage with increased accuracy [25].

Emerging biomarkers, such as serum potassium levels, molecules involved in extrinsic apoptosis (Fas and soluble Fas ligand), cathepsin D and zonulin, provide additional insights into monitoring the progression and severity of liver injury.

Elevated serum uric acid reflects increased fructose intake and is associated with insulin resistance, while Vitamin D’s anti-inflammatory and anti-fibrotic properties suggest a potential hepatoprotective role [25].

Additional tests to rule out other specific liver diseases would include tests for celiac disease (total IgA and tissue transglutaminase), viral hepatitis [71], testing of serum ceruloplasmin levels and possibly a 24 h urinary copper test if Wilson’s disease (WD) is suspected [10]. It is also important to rule out autoimmune hepatitis and therefore it is recommended to determine anti-nuclear antibodies (ANAs), liver-renal microsomal antibodies, anti-smooth muscle cell antibodies and total IgG. Testing for alpha-1 antitrypsin deficiency is also recommended [77], as well as an enzymatic test for lysosomal acid lipase activity if the presentation raises the suspicion of LAL-D [10].

The first-line tests used in the diagnosis of NAFLD in children are tests that monitor liver function: ALT, AST, CPK, GGT, conjugated and unconjugated bilirubin, protein electrophoresis, serum albumin, prothrombin time, and partial thromboplastin time. The second line of investigation aims to rule out more common liver or systemic diseases that have similar manifestations and it consists of: viral markers (HAV, HBV, HCV), hepatotropic viruses serology (e.g., EBV, CMV), ceruloplasmin, serum copper, ANA, SMA, LKM, LC1, anti-SLA, total IgG, serum α1 antitrypsin, EMA, tTgasi IgA, deamidated AGA, IgA. The third line consists of tests or imaging procedures that exclude or rule out other conditions that are part of the differential diagnosis of NAFLD: urinary copper, genetic and metabolic enlarged screening, fecal elastase, other hepatic imaging techniques (MRI, CT, etc.), liver biopsy, etc.

## 9. Imaging Methods

Liver ultrasonography is the first-line imaging method for assessing hepatic steatosis, as it is easily accessible, non-invasive, and low-cost. It allows for the estimation of the degree of lipid accumulation in the liver based on specific characteristics, but its sensitivity decreases in the presence of less than 30% infiltration or in patients with severe obesity (BMI ≥ 40) [3].

Liver ultrasonography is a safe method, but it has limitations in detecting hepatic steatosis when it affects less than 30% of hepatocytes, due to the difficulty in differentiating the echogenicity of the liver from that of the renal parenchyma [24].

Imaging methods used to assess liver fibrosis include transient elastography, shear wave elastography, and magnetic resonance elastography [74].

Vibration-controlled transient elastography (VCTE; FibroScan) uses transient elastography to assess liver fibrosis and shows a good correlation with histologically confirmed fibrosis in both adults and children. Recent studies support the reliability of transient elastography in differentiating stages of fibrosis in pediatric patients, although it provides more accurate results in autoimmune and post-transplant liver diseases compared to NAFLD, where fibrosis must be excluded with caution [3]. It provides measurements of liver stiffness (LSM, expressed in kilopascals [kPa]), which reflects the degree of fibrosis, and controlled attenuation parameter (CAP, expressed in decibels per meter), estimating the degree of hepatic steatosis. CAP measurements have been shown to correlate significantly with steatosis in children with NAFLD [4].

Magnetic resonance elastography (MRE) stands out as a promising tool for differentiating between simple steatosis and nonalcoholic steatohepatitis (NASH), being able to distinguish advanced fibrosis (stages 3–4) from mild fibrosis (stages 0–2) with a sensitivity of 86% and a specificity of 91% [25].

Magnetic resonance (MR) offers advanced methods for the assessment of hepatic steatosis and fibrosis, but its widespread use is limited by high costs and lack of cost-effectiveness, even in the context of modern technologies that allow for rapid and reproducible measurements. Modern MR spectroscopy techniques allow the determination of intracellular water and lipid content, and a triglyceride/liver water ratio above 0.5 can define the presence of steatosis [25].

## 10. Non-Invasive Indices

Serum biomarkers for liver fibrosis, such as the NAFLD fibrosis score (NFS) and Fibrosis-4 Index (FIB-4), extensively studied in the adult NAFLD population, do not have acceptable parameters to be applied to children [78].

There are numerous markers for fibrosis studied in recent years in children, such as tumor necrosis factor-α, interleukin-6, leptin, adiponectin, fibroblast growth factor and cytokeratin-18, but they do not seem to have the sensitivity and specificity needed for the accurate diagnosis of NAFLD [76].

The values of the fatty fraction of liver proton density, measured by magnetic resonance, correlate well with the grade of steatosis confirmed by histology and may be useful in the early detection of the disease [76].

## 11. Liver Biopsy

Considered the gold standard, liver biopsy is indicated in cases with clinical suspicion of advanced involvement, to exclude other etiologies or before pharmacologic therapy is instituted [24].

The main histologic changes associated with NAFLD in children include the presence of hepatic steatosis, hepatocyte ballooning, inflammation and the development of hepatic fibrosis [3].

NAFLD, in both children and adults, is histologically characterized by the presence of macrovesicular steatosis in at least 5% of hepatocytes, in the absence of other known causes that could lead to fat accumulation in the liver. However, there are significant differences in liver histologic appearance between the two age groups [77]. In adults, lipid accumulation predominantly starts in the perivenular zone (acinar zone 3), whereas in children steatosis is usually localized to the periportal zone (zone 1) or has an azonal pattern. The hepatic inflammatory infiltrate is variable and may include lymphocytes, histiocytes and Kupffer cells and is present at both lobular and portal levels [3].

Hepatocyte ballooning is a defining histologic feature in nonalcoholic steatohepatitis (NASH) and is associated with an increased risk of progression to advanced forms of the disease. This cellular alteration triggers tissue remodeling pathways and fibrogenesis by recruiting cell populations atypical for the healthy liver. Activation of these mechanisms favors the progression of the lesions to liver fibrosis and, over time, cirrhosis [4].

Two main scoring systems are used for the histologic evaluation of NAFLD and NASH: the Brunt score and the NAFLD Activity Score (NAS). The former semi-quantitatively assesses the degree of macrovacuolar steatosis, bloating, lobular and portal inflammation, classifying the activity into mild, moderate and severe. The second system generates a composite numerical score of histologic activity, calculated by summing the individual scores for steatosis, lobular inflammation, and bloating, with a scale from 0 to 8. Lower scores (0–2) suggest the absence of NASH, intermediate scores (3–4) are considered borderline, and higher values (≥5) are suggestive of the presence of disease. Both systems also include a staging classification of liver fibrosis, from mild perisinusoidal fibrosis (stage 1) to liver cirrhosis (stage 4) [3].

Liver biopsy should be taken into consideration early in patients with a family history of NASH, hepatosplenomegaly, associated comorbidities, expansile hypothalamic lesions, significantly elevated transaminase values or serum markers indicative of fibrosis [24].

## 12. Treatment

### 12.1. Lifestyle Interventions

The first-line therapeutic strategy in pediatric NAFLD consists of lifestyle interventions focused on normalizing body weight through a balanced diet and regular physical activity. Although dietary supplements such as omega-3 fatty acids and probiotics have been investigated in clinical trials, the existing data are not yet sufficient to support their routine use and further rigorous investigations are needed to establish their efficacy and safety in current pediatric practice [73].

Diet and exercise improve insulin sensitivity and glucose and lipid metabolism, and long-term sustained changes (at least 24 months) may have histologic benefits in the liver [24].

The active involvement of the family and the pediatrician is essential for the success of the intervention. The American Academy of Pediatrics (AAP) recommends a tiered approach. The first stage is prevention, aimed at normal-weight children, and includes eating at least five servings of fruits and vegetables daily, avoiding sugar-containing beverages, getting at least one hour of daily physical activity, reducing screen time to less than two hours a day, and eliminating TV in the bedroom. The second stage, called “Prevention Plus”, applies to overweight children and involves 5–6 family meals per week without restrictive behaviors, trying to maintain weight maintenance with BMI reduction as the child grows. Physical activity is structured with 1 h of at least moderate physical activity per day and 20 min of vigorous activity 3 times per week and encouragement of family activities. The third stage targets children with obesity (BMI ≥ 95th percentile) and involves complex multidisciplinary management which includes the family doctor or pediatrician, a nutritionist, a psychologist and other specialties, as appropriate [24]. The goal is weight maintenance or gradual weight loss until their BMI is <85th percentile and the indicated measures are limiting television (0 h for children < 2 years and <2 h for children > 2 years), limiting consumption of sugar-containing beverages, encouraging family meals, decreasing portion sizes, removing television from the main sleeping area, eating breakfast daily, limiting eating out [79].

The Mediterranean diet, low in saturated fat and animal protein and high in fiber, antioxidants and monosaturated fatty acids, is a healthy dietary option that prevents obesity and the development of liver steatosis [80].

A systematic review and meta-analysis showed that supervised physical training, both aerobic and resistance training, at vigorous or moderate to vigorous intensities, reduces hepatic fat content, which is an effective strategy in the prevention and treatment of NAFLD/MASLD in children and adolescents [81].

A randomized study conducted on children with NAFLD who received recommendations regarding physical activity and diet, without other drug interventions, concluded that 23% showed worsening fibrosis and 18% progressed to NASH. Although fibrosis improvement was present in 34% of children, this study indicates the need to identify risk factors that contribute to the lack of response to lifestyle intervention therapy [82].

Lifestyle interventions and specific diets for the treatment of obesity in NAFLD should involve the whole family and be combined with cognitive-behavioral techniques for better results. Strategies should be tailored to the patient’s environment and needs and should involve the community (parents, teachers, friends, relatives, classmates) [22].

### 12.2. Pharmacological Approaches

Considerable progress is being made in identifying therapeutic targets for the treatment of NASH, beyond the current recommendations regarding the modification of the lifestyle. Pharmacological approaches target multiple mechanisms involved in the pathogenesis of the disease, such as: cell apoptosis (the use of antioxidants such as vitamin E), metabolic dysfunctions (GLP-1 receptor agonists such as liraglutide), gut-liver interaction (FGF-19 agonists such as NGM282), profibrotic processes (LOXL2 inhibitors such as Simtuzumab), as well as inflammation (CCR2/CCR5 chemokine receptor antagonists such as Cenicriviroc) [4]. Although data from the literature on adults with NAFLD report promising effects when using these molecules, there is currently no evidence to support their use in children.

Vitamin D supplementation is recommended in patients with NAFLD, because studies have suggested that vitamin D deficiency favors the acceleration of liver fibrogenesis [83].

Also, children treated with vitamin E have shown significant improvements in NASH resolution compared to the placebo, due to the antioxidant effect [5].

Decreased choline intake is associated with worsening fibrosis, therefore choline supplementation is recommended in choline deficient patients on long-term parenteral nutrition. Studies have also confirmed the restoration of insulin sensitivity and the anti-inflammatory effect of a diet enriched with the omega-3 fatty acids DHA and eicosapentaenoic acid in patients with NAFLD [84].

Gut microbiota is involved in lowering blood pressure and blood cholesterol levels, as well as the significant regulation of the energy balance, thus studies have shown that the modification of gut microbiota with prebiotics and probiotics is beneficial for weight reduction [85,86].

The most studied strains were *Lactobacillus rhamnosus*, which reduced liver inflammation, improved lipid metabolism and increased the production of certain anorexigenic gut hormones [85] and *Lactobacillus GG*, which caused a significant decrease (up to normalization) in serum ALT values [86,87]. In a triple-blind randomized study of 64 obese children with NAFLD who were given probiotic capsules (containing *Lactobacillus acidophilus*; *Bifidobacterium lactis*; *Bifidobacterium bifidum*; *Lactobacillus rhamnosus*) or placebo, a significant decrease in waist circumference, transaminase levels, triglycerides, cholesterol, low-density lipoproteins, and an improvement in liver structure on ultrasound scans were observed at 12 weeks in the intervention group compared to the placebo group. Further studies and long-term follow-up of patients are needed to establish the safety profile of this therapeutic option [88].

### 12.3. Medical Therapy

The only drugs currently approved by the Food and Drug Administration (FDA) for the treatment of pediatric obesity are Orlistat, approved for children over 12 years of age, and Sibutramine, approved for children over 16 years of age.

Sibutramine has the effect of reducing appetite, as it is a non-specific inhibitor of serotonin, norepinephrine and dopamine reuptake. Because of its vasoconstrictive side-effect, it excludes its use in children with hypertension.

Orlistat inhibits endoluminal lipase and has numerous side effects, among which it leads to chronic kidney disease due to secondary hyperoxaluria. This is why patients should follow a low-oxalate, calcium-rich diet with increased daily water intake [24].

Insulin resistance improved by Metformin has been associated in some studies with reduced steatosis, but the results are controversial [87]. Studies conducted on adults have reported a significant improvement of NASH in patients using Liraglutide, a glucagon-like peptide-1 (GLP-1) agonist, but further studies are needed. It is not used in pediatric practice because of its injectable administration.

Results obtained in adults with NASH demonstrated the positive effect of pioglitazone on improving steatosis and lobular inflammation in patients with NASH, but it has not been authorized for use in children due to cardiovascular side effects and the risk of bladder cancer [89].

Cysteamine is a precursor for glutathione synthesis that has antioxidant properties and an insulin-sensitizing effect by regulating adiponectin levels, which has reduced transaminase levels in children with NAFLD without reducing their body mass index [90].

Resveratrol is a plant-derived polyphenol that reduces liver inflammation and improves lipid metabolism in adults [91], while pentoxifylline, which is a phosphodiesterase inhibitor that decreases TNF-α gene transcription [92], and obeticholic acid improves liver histology in adults with NAFLD [89].

Ezetimibe is a drug that selectively inhibits cholesterol absorption in the small intestine by binding to the brush border and has been shown to improve liver histology in adult patients, with an increased safety profile when combined with 3-hydroxy-3-methylglutaryl coenzyme A reductase inhibitors [93].

The clinical potential of silibinin has been demonstrated in several randomized studies in adults with histologically documented NAFLD, where treatment was associated with improvement in liver enzymes and liver histology, without increase in body weight [24].

Pediatric studies, similarly to adult studies, have not demonstrated the efficacy of ursodeoxycholic acid, a well-known bile acid with antioxidant, immunomodulatory, antiapoptotic, and cytoprotective functions, in the treatment of NAFLD, either as a single agent or in combination (with vitamin E, with or without lifestyle interventions) [22].

Studies in adults and children with growth deficiency and NASH have shown that growth hormone (GH) replacement therapy improves serum transaminase levels [94].

The development of drugs for the treatment of NASH, both in adults and children, is of great interest, but many of these drugs have not been proven effective in reducing liver fibrosis, or the studies are in various clinical stages that require follow-up. Clinical studies in pediatric patients are difficult to conduct, as many of the drugs are not administered at young ages due to severe adverse reactions, high costs or inappropriate routes of administration.

### 12.4. Surgical Therapies

There are numerous surgical techniques used in adults that aim to reduce gastric volume, food absorption and induce early satiety (intragastric balloons, Roux-en-Y gastric bypass, reversible adjustable gastric banding, laparoscopic sleeve gastrectomy), or that create gastric stasis by providing a feeling of early satiety, such as truncal vagotomy [95]. Considering the numerous complications, as well as the implications on growth, these interventions are taken into consideration in adolescent children with morbid obesity, or when they have associated diabetes, OSA or when there is impairment in the quality of life and daily activities [95], as part of multidisciplinary treatment.

In a study of 19 obese adolescents who underwent the endoscopic insertion of a duodenojejunal bypass liner, a decrease in BMI at 1 year was achieved, from 41.1 kg/m^2^ to 37.2 kg/m^2^, along with a decrease in ALT levels and improvement in hepatic steatosis [96].

#### Current Limitations and Future Research Directions

Currently, the actual prevalence of pediatric NAFLD in obesity is unknown. Identifying risk factors and optimizing screening practices will help determine an accurate estimate of the impact of NAFLD on children’s health and the socioeconomic impact of this chronic disease, which requires early intervention to avoid complications. Current data do not allow us to accurately determine the progression of the disease, therefore future studies are needed on a large number of patients of different ages, genders, and geographical areas, monitoring BMI, transaminases, liver biopsy, MRI, or associated comorbidities.

Currently, the therapeutic strategy for NAFLD remains lifestyle intervention through diet and increased physical activity, while research into the complex pathophysiology of NAFLD is uncovering new therapeutic targets.

## 13. Conclusions

Due to the exponential increase in childhood obesity, there is a risk of inflammation, liver fibrosis, and pathological angiogenesis. The pathogenesis of NAFLD is complex and represents a combination of genetic and environmental factors, with an important role attributed to the gut microbiota. Further studies are needed to understand the natural history, pathophysiology, assessment, and treatment of children with NAFLD in order to optimize the care of children with the most common cause of chronic liver disease. There is no effective treatment for NAFLD in children; consequently, lifestyle interventions (diet and exercise) represent a real challenge due to lack of compliance. Considerable progress is being made in identifying pharmacological therapeutic targets for the treatment of NAFLD in those who do not adhere to lifestyle modification recommendations based on understanding the mechanisms that initiate and maintain intestinal inflammation, the role of genetic markers and the intestinal microbiome in NAFLD.

Thus, although early identification of NAFLD is essential to prevent complications, including progression to severe liver disease, the most important thing remains the prevention of obesity in children.

## Figures and Tables

**Table 1:** Prevalence of pediatric NAFLD by gender in the general population and in children with obesity: comparison between two meta-analyses conducted in 2024 and 2015, respectively.

	Prevalence % in the General Population (2024)	Prevalence % of Obese Population (2024)	Prevalence % in the General Population (2015)	Prevalence % of Obese Population (2015)
Overall prevalence of NAFLD	13	47	2.3	36.1
Female	10	39	6.3	21.8
Male	15	54	9	35.3

## Data Availability

Data is contained within the article.

## Abbreviations

The following abbreviations are used in this manuscript:

NAFLD	Non-alcoholic fatty liver disease
MASLD	Metabolic dysfunction-associated steatotic liver disease
NASH	Non-alcoholic steatohepatitis
BMI	Body mass index
TNF-alpha	Tumor necrosis factor-alpha
IL-6	Interleukin 6
CVD	Cardiovascular disease
DNA	Deoxyribonucleic acid
mRNA	Messenger ribonucleic acid
HSCs	Hepatic stellate cells
TG	Triglyceride
LPL	Lipoprotein lipase
FFA	Free fatty acids
ApoC-III	Apolipoprotein C-III
NAD	Nicotinamide adenine dinucleotide
FAD	Flavin adenine dinucleotide
CYP4A	Cytochrome P450 4A
ROS	Cytotoxic reactive oxygen species
NFκB	Nuclear factor pathway κB
MOMP	Mitochondrial outer membrane permeabilization
PNPLA3	Adiponutrin/patatin-like phospholipase domain-containing 3
ALT	Alanine aminotransferase
AST	Aspartate aminotransferase
LAL	Lysosomal acid lipase
GCKR	Glucokinase regulatory protein
APOC3	Apolipoprotein C3
TM6SF2	Human transmembrane 6 superfamily 2
ChREBP	Carbohydrate response element-binding protein
GLUT5	Glucose transporter protein type-5
ATP	Hepatic adenosine triphosphate
VLDL	Very-low-density lipoprotein
PRRs	Pattern recognition receptors
TLRs	Toll-like receptors
NOD-like receptors	Nucleotide-binding oligomerization domain-like receptors
SCFAs	Short-chain fatty acids
ChREBP	Hepatic carbohydrate response element-binding protein
BCFAs	Branched-chain fatty acids
NO	Nitric oxide
H2S	Hydrogen sulfide
FXR	Farnesoid X receptor
NLRP3	Pyrin domain-containing protein 3
SEC	Sinusoidal endothelial cells
ADH	Insulin-dependent alcohol dehydrogenase
OSA	Obstructive sleep apnea
GOAT	Ghrelin-ghrelin O-acyltransferase
HFD	High-fat diet
PCOS	Polycystic ovary syndrome
MRI	Magnetic resonance imaging
GGT	Gamma-glutamyl transferase
BA	Serum bile acid
HOMA-IR	Homeostatic Model Assessment for Insulin Resistance
CK-18	Cytokeratin 18
ANAs	Anti-nuclear antibodies
TGR	G protein-coupled receptor
CAP	Controlled attenuation parameter
NFS	NAFLD fibrosis score
FIB-4	Fibrosis-4 Index
AAP	American Academy of Pediatrics
GLP-1	Glucagon-like peptide
LOXL2	Lysyl Oxidase-like 2
FDA	Food and Drug Administration
